# Long non-coding RNA PRR7-AS1 promotes osteosarcoma progression via binding RNF2 to transcriptionally suppress MTUS1

**DOI:** 10.3389/fonc.2023.1227789

**Published:** 2023-11-16

**Authors:** Gu Chen-Xi, Xu Jin-Fu, Huang An-Quan, Yu Xiao, Wu Ying-Hui, Li Suo-Yuan, Shen Cong, Zou Tian-Ming, Shen Jun

**Affiliations:** ^1^ Department of Orthopedic Surgery, The Affiliated Suzhou Hospital of Nanjing Medical University, Suzhou Municipal Hospital, Gusu School, Nanjing Medical University, Suzhou, China; ^2^ State Key Laboratory of Reproductive Medicine, Department of Histology and Embryology, Nanjing Medical University, Nanjing, China; ^3^ State Key Laboratory of Reproductive Medicine, Center for Reproduction and Genetics, The Affiliated Suzhou Hospital of Nanjing Medical University, Suzhou Municipal Hospital, Gusu School, Nanjing Medical University, Suzhou, China

**Keywords:** LncRNA PRR7-AS1, RNF2, osteosarcoma, histone modification, MTUS1 LncRNA PRR7-AS1, MTUS1

## Abstract

**Introduction:**

Osteosarcoma is a common bone malignant tumor in adolescents with high mortality and poor prognosis. At present, the progress of osteosarcoma and effective treatment strategies are not clear. This study provides a new potential target for the progression and treatment of osteosarcoma.

**Methods:**

The relationship between lncRNA PRR7-AS1 and osteosarcoma was analyzed using the osteosarcoma databases and clinical sample testing. Cell function assays and tumor lung metastasis were employed to study the effects of PRR7-AS1 on tumorigenesis *in vivo* and *in vitro*. Potential downstream RNF2 of PRR7-AS1 was identified and explored using RNA pulldown and RIP. The GTRD and KnockTF database were used to predict the downstream target gene, MTUS1, and ChIP-qPCR experiments were used to verify the working mechanismy. Rescue experiments were utilized to confirm the role of MTUS1 in the pathway.

**Results:**

Deep mining of osteosarcoma databases combined with clinical sample testing revealed a positive correlation between lncRNA PRR7-AS1 and osteosarcoma progression. Knockdown of PRR7-AS1 inhibited osteosarcoma cell proliferation and metastasis in vitro and in vivo. Mechanistically, RNA pulldown and RIP revealed that PRR7-AS1 may bind RNF2 to play a cancer-promoting role. ChIP-qPCR experiments were utilized to validate the working mechanism of the downstream target gene MTUS1. RNF2 inhibited the transcription of MTUS1 through histone H2A lysine 119 monoubiquitin. Rescue experiments confirmed MTUS1 as a downstream direct target of PRR7-AS1 and RNF2.

**Discussion:**

We identified lncRNA PRR7-AS1 as an important oncogene in osteosarcoma progression, indicating that it may be a potential target for diagnosis and prognosis of osteosarcoma.

## 1 Introduction

Osteosarcoma (OS) is a common malignant bone tumor that mainly affects adolescents or children under the age of 20 years (1). OS accounts for approximately 5% of pediatric tumors, and it is a highly malignant tumor in children (2). OS has poor prognosis, and lung metastasis usually occurs within months. It has been reported that the survival of OS patients after amputation ranges from 5 to 20% (3). At present, surgery combined with neoadjuvant radiotherapy and chemotherapy is preferred to treat OS (4). Early diagnosis and timely treatment significantly improve the survival of OS (5). Therefore, it is urgent to elucidate the pathogenesis of OS and to identify more effective therapeutic targets to improve the prognosis of OS.

Long non-coding RNAs (lncRNAs) are non-coding RNAs exceeding 200 bp in length. LncRNAs are unable to directly encode proteins, but they are vital regulators in biological activities via interacting with proteins, DNA and RNA (6). Serving as oncogenes or tumor suppressor genes (7), lncRNAs have been highlighted for their involvement in the development of cancer, including prostate, breast, lung and liver cancers, through chromatin modification, transcriptional regulation and post-transcriptional regulation (8–10). Ding et al. (11) demonstrated that lncRNA CRNDE stimulates the growth of OS through the Wnt/β-catenin signaling pathway. Through downregulating microRNA-765, LINC00511 promotes the proliferation and migration of OS cells (12). Thus, the specific role of lncRNAs in the development of OS and the underlying mechanism should be further explored.

A novel oncogene PRR7-AS1 has been reported that is highly expressed in colorectal cancer (13) and hepatocellular cancer (14). In this study, we showed that lncRNA PRR7-AS1 was upregulated in OS tissues through bioinformatics analysis. The upregulation of PRR7-AS1 was further validated in clinical samples. Subsequently, *in vitro* and *in vivo* experiments demonstrated that PRR7-AS1 promoted the proliferation and migration of OS through binding RNF2, which further inhibited the transcription of the downstream target, MTUS1. The present study identified the oncogenic role of PRR7-AS1 in OS, suggesting its potential application in the clinical treatment of OS.

## 2 Materials and methods

### 2.1 Bioinformatics analysis

The GSE126209 dataset, contains RNA sequencing data from OS and normal tissues, was downloaded from Gene Expression Omnibus (GEO, https://www.ncbi.nlm.nih.gov/geo/). The FPKM values were transformed into TPM values and normalized by log_2_(value+1) to further analysis. Afterwards, paired differential expression was analyzed using the R package limma and volcanic maps were draw by ggplot2 (|FC | > 1, and adj.p < 0.05).

Clinical and gene expression data of OS patients were obtained from the Tumor Alterations Relevant for Genomics-driven Therapy (TARGET) database. Patient samples (n=85) were divided into high and low groups based on the median of expression values, and survival curves were plotted by the “survival” package in R.

Putative RNF2 targets were predicted using the Gene Transcription Regulation Database (GTRD, http://gtrd.biouml.org) and the KnockTF Database (http://www.licpathway.net/KnockTF/index.php).

### 2.2 Sample collection

OS and adjacent non-tumoral bone tissues were collected from patients who were surgically treated at the Suzhou Municipal Hospital of Nanjing Medical University. Clinical samples were surgically collected, immediately fixed, dehydrated and embedded in paraffin. Written informed consent was obtained prior to sample collection, and this study was approved by the Ethics Committee of Suzhou Municipal Hospital.

### 2.3 Fluorescence *in situ* hybridization analysis

A specific FISH probe targeting PRR7-AS1 was designed and synthesized by Ribobio Biotechnology (Guangzhou, China). The hybridization was performed in OS tissue and paired adjacent non-tumoral tissues as previously reported (15). All images were analyzed on a confocal laser scanning microscope (LSM 810, Carl Zeiss, Oberkochen, Germany) (16).

### 2.4 Cell culture

The human osteosarcoma cell lines, 143B and U2OS, were obtained from the Institute of Biochemistry and Cell Biology of the Chinese Academy of Sciences (Shanghai, China). U2OS cells were cultured in Dulbecco’s modified Eagle’s medium (DMEM) (Gibco, NY, USA) supplemented with 10% fetal bovine serum (FBS) (ExCell Bio, New Zealand) and 1% penicillin/streptomycin (NCM Biotech, Suzhou, China), and 143B cells were cultured in Eagle’s Minimum Essential Medium (EMEM) (Gibco) supplemented with 82% minimal essential medium (MEM), 15% FBS, 1% GlutaMAX, 1% sodium pyruvate and 1% penicillin/streptomycin. Cells were cultured in a humidified incubator with 5% CO2 and 95% air at 37°C.

### 2.5 Cell transfection

The small interfering RNAs (siRNAs) targeting PRR7-AS1, RNF2, MTUS1 and negative control and pcDNA3.1-PRR7-AS1 were designed by GenePharma (Shanghai, China) and transfected into cells using Lipofectamine 2000 (Invitrogen, USA) as described previously (17). After 48 h, cells were collected for subsequent experiments. The siRNA sequences used in the present study were shown on **Table S1**.

### 2.6 RNA extraction and real-time quantitative PCR

Total RNA was extracted from cells using the RNA isolater Total RNA Extraction Reagent (Vazyme, Nanjing, China), which was reverse transcribed into cDNA using the HiScript III RT SuperMix for qPCR (+gDNA wiper) kit (R323-01, Vazyme) as described previously (18, 19). RT-qPCR was performed using SYBR qPCR SuperMix Plus (Novoprotein Scientific Inc., Shanghai, China) and an Applied Biosystems 7500 Real Time PCR System. The primers were shown on **Table S2**.

### 2.7 Western blot

Osteosarcoma cells transfected with si-NC/si-PRR7-AS1 were lysed using a radioimmunoprecipitation assay (RIPA, Beyotime, Nantong, China) containing 1% protease inhibitor phenylmethylsulfonyl fluoride (PMSF) (17). After quantified using a bicinchoninic acid (Beyotime) kit, the protein samples were separated by SDS-PAGE (sodium dodecyl sulfate-polyacrylamide gel electrophoresis) and transferred onto polyvinylidene difluoride (PVDF) membranes. The membranes were blocked with 5% skim milk, and then incubated with the anti-RNF2 antibody (Proteintech, Wuhan, Hubei, China) and anti-Tubulin (Beyotime) at 4°C overnight. Secondary antibodies combined with horseradish peroxidase at room temperature were incubated and band signals were visualized by an enhanced chemiluminescent substrate and quantified by Image-Pro Plus (Media Cybernetics, San Diego, CA, USA).

### 2.8 Cell proliferation and migration assay

Cell Counting Kit-8 (CCK8) assay: cells were seeded into 96-well plate with 2.5×10^3^ cells/well. CCK8 (Beyotime) was added into each well at 0, 24, 36, 72, 96 h. Cell viability was detected by measuring the optical density at 450 nm (OD450) using a microplate reader (Bio-Rad Model 680, Richmond, CA, USA).

Colony formation assay: cells were seeded into six-well plate with 1.0×10^3^ cells/well and cultured for 14 days. Fresh medium was replaced every 5 days. After 14 days, culture medium was removed, and cells were fixed with methanol and stained with 0.1% crystal violet (Beyotime). Visible colonies were imaged and counted.

Transwell assay: transwell chambers (8 μm pore size; Millipore, Billerica, MA, USA) were added into 24-well plate. Cells at a density of 2.5 × 10^4^ cells/well in 300 µl of serum-free medium were seeded in the upper chamber, and 700 µl of medium containing 10% FBS was added in the bottom chamber. After incubation for 48 h at 37°C, the cells migrated to the bottom were fixed with methanol and stained with 0.1% crystal violet. Finally, five randomly selected fields per sample were imaged using a light microscope for counting migratory cells.

### 2.9 *In vivo* assay

Four-week-old athymic BALB/c nude mice were habituated in a specific pathogen-free (SPF) environment. Animal experiments were approved by the Ethics Committee of Animal Experiments of Nanjing Medical University. Briefly, 100 µl of suspended 143B cells transfected with sh-PRR7-AS1 or sh-NC at 3×10^7^ cells were subcutaneously injected into the mouse axillary region (17). Tumor growth was regularly recorded. After 2 weeks, mice were sacrificed, and OS tissues were collected. The tumor volume was calculated using the following formula: tumor volume (mm^3^) = 0.5 × length (mm) × width^2^ (mm^2^).

For the *in vivo* cell metastasis assay, 100 µl of suspended 143B cells transfected with sh-PRR7-AS1 or sh-NC at 6×10^7^ cells was injected into the mouse tail vein. After 2 months, mice were sacrificed, and lung tissues were collected. The number of metastatic lesions in the lung was counted.

Tumor tissues and lung metastasis lesions collected from mice were fixed with 4% paraformaldehyde and dehydrated in gradient concentrations of ethanol. After permeabilization in xylene, sections were embedded in paraffin, sliced into 5-µm-thick sections, deparaffinized with xylene and rehydrated in gradient concentrations of ethanol. Lung metastasis sections were stained with hematoxylin and eosin (H&E) for pathological examination.

### 2.10 Immunofluorescence

After deparaffinization and rehydration of paraffin-embedded tissues, antigen retrieval was performed by boiling samples in 10 mM sodium citrate buffer (pH 6.0). The sections were blocked with 1% bovine serum albumin (BSA) in PBS and incubated with primary antibodies, and the sections were then incubated with secondary antibodies. Cell nuclei were stained with DAPI (Beyotime), and images were acquired using a Zeiss laser confocal microscope (LSM 810) as previously described (20, 21). The following antibodies were used: mouse monoclonal anti-Ki67 antibody (Abcam, ab238020, 1:100), mouse monoclonal anti-E-cadherin antibody (Abcam, ab231303, 1:100), mouse monoclonal anti-N-cadherin antibody (Abcam, ab76057, 1:100) and mouse monoclonal anti-vimentin antibody (Abcam, ab20346, 1:100).

Isolated and cultured cells were fixed with 4% paraformaldehyde for 20 min. Next, the cells were treated with 0.2% triton for 20 min and blocked with 1% BSA in PBS. Then the slides containing target cells were incubated with primary and Alexa-Fluor secondary antibodies (Thermo Scientific, Waltham, USA) orderly. In addition, the cells were stained with DAPI and observed under a fluorescence microscope. Sections were analyzed under a confocal laser-scanning microscope.

### 2.11 RNA pull-down

PRR7-AS1 was transcribed using the Ribo™ RNAmax-T7 Biotin Labeling Transcription Kit (Ambio Life). The kit used the Biotin RNA Labeling Mix as a substrate and utilized a DNA template containing the T7 promoter to synthesize RNA complementary to the antisense strand in the DNA template starting downstream of the T7 promoter. Then PRR7-AS1 was purified with the RNeasy Plus Mini Kit (Qiagen) and treated with RNase-free DNase I (Qiagen). Subsequently, transcribed PRR7-AS1 was biotin-labeled with the Biotin RNA Labeling Mix (Ambio Life). RNA pull-down was then performed by PierceTM Magnetic RNA-Protein Pull-Down Kit according to the manufacturer’s instructions (Thermo Scientific Pierce) and as previously described (22). The RNA-protein complexes were then subjected to mass spectrometry analysis, as previously described (23–25).

### 2.12 Chromatin immunoprecipitation-qPCR

ChIP was performed using the EZ-Magna ChIPTM A/G Chromatin Immunoprecipitation Kit (Millipore) as previously described (26). Briefly, transfected OS cells were lysed in 1% methanol and sonicated, which resulted in 500-bp DNA fragments. Cell lysate was incubated with the anti-RNF2 antibody, anti-H2AK119Ub antibody and anti-IgG. The chromatin supernatant was then incubated in 20 μl of protein A/G MagBeads at 4°C overnight. The protein-DNA complex was eluted and purified, and the obtained DNA samples were subjected to RT-qPCR. The primers used for ChIP-qPCR were follows:

MTUS1-F, 5’-AGACTGCGAATCAGCCCTTC-3’
MTUS1-R, 5’-TGCAGAATTATCAGGGCGGAA-3’

### 2.13 RNA immunoprecipitation

RIP was performed using the Magna RIPTM RNA-Binding Protein Immunoprecipitation Kit (Millipore) as previously described (16). Briefly, OS cells were lysed in RIP lysis buffer, and 100 µl of cell lysate was incubated with the anti-RNF2 antibody and anti-IgG. A protein–RNA complex was captured and digested with 0.5 mg/ml proteinase K containing 0.1% SDS. The magnetic beads were repeatedly washed with RIP washing buffer to remove non-specific adsorption. Finally, the extracted RNA was subjected to RT-qPCR.

### 2.14 Statistical analysis

All data are expressed as mean ± standard deviation ( $\bar{x}\pm \text{s}$ ). GraphPad Prism 9.0 (GraphPad Software, CA, USA) was used for statistical analyses. Differences between two groups were compared by Student’s t−test, and those among three or more groups were compared by one−way analysis of variance (ANOVA). Overall survival was estimated by the Kaplan–Meier method, and the log-rank test was employed to evaluate differences. P<0.05 was considered as statistically significant.

## 3 Results

### 3.1 PRR7-AS1 is upregulated in OS tissues and predicts poor prognosis

**Figure 1:**
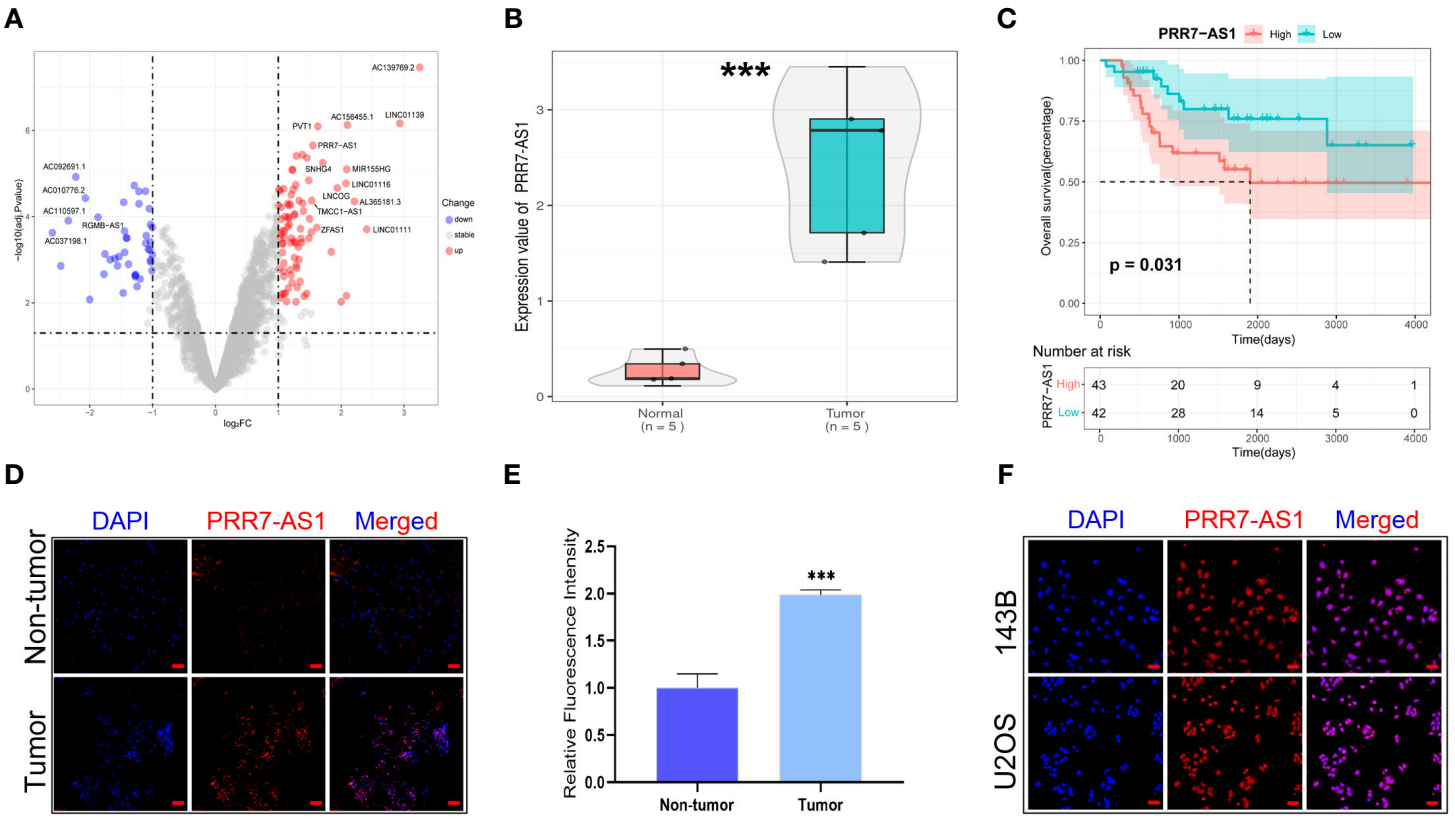
Relative PRR7-AS1 expression in osteosarcoma (OS) tissues and cell lines as well as its clinical significance. **(A)** Paired differentially expressed lncRNAs in OC and normal tissues based on GEO datasets (n=5) showed by volcanic map. **(B)** Relative expression of PRR7-AS1 in OC and normal tissues, n = 5. ***P < 0.001. **(C)** Kaplan–Meier curves for overall survival in OS patients (n=85). **(D)** A FISH assay was used to examine the expression and location of lncRNA PRR7-AS1 in OS tissues and paired non-tumor tissues. scale bars: 50 mm. **(E)** Statistical data are shown. **(F)** A FISH assay was used to examine the location of PRR7-AS1 in 143B and U2OS, scale bars: 50 mm. ***P < 0.001.

A total 19 lncRNAs were differentially expressed in osteosarcoma tissues (n=5) compared with paired normal tissues based on the GEO database (GSE126209) (|FC| > 1.0 and adj.p < 0.01) (**Figure 1A**), and 2 of them (PVT1 and PRR7-AS1) had a significant effect on the overall survival of OS patients (n=85) based on the TARGET database (**Figure S1**). It has been found that PVT1 (plasmacytoma variant translocation 1) could promote human OS malignant biological behaviors (27), but the role of PRR7-AS1 in osteosarcoma remains unknown.

PRR7-AS1 was significantly upregulated in OS tissues (n=5) (**Figure 1B**), and high PRR7-AS1 expression was associated with poor prognosis in OS patients (**Figure 1C**). Afterwards, the FISH assay verified the overexpression of PRR7-AS1 in OS tissues compared to non-tumor samples (**Figures 1C, D**), and it also demonstrated that PRR7-AS1 was mostly expressed in the nucleus (**Figures 1C–F**). These results suggested that PRR7-AS1 is upregulated in OS and mainly involved in functional regulation in the nucleus.

### 3.2 PRR7−AS1 promotes the proliferation and migration of OS *in vitro*

**Figure 2:**
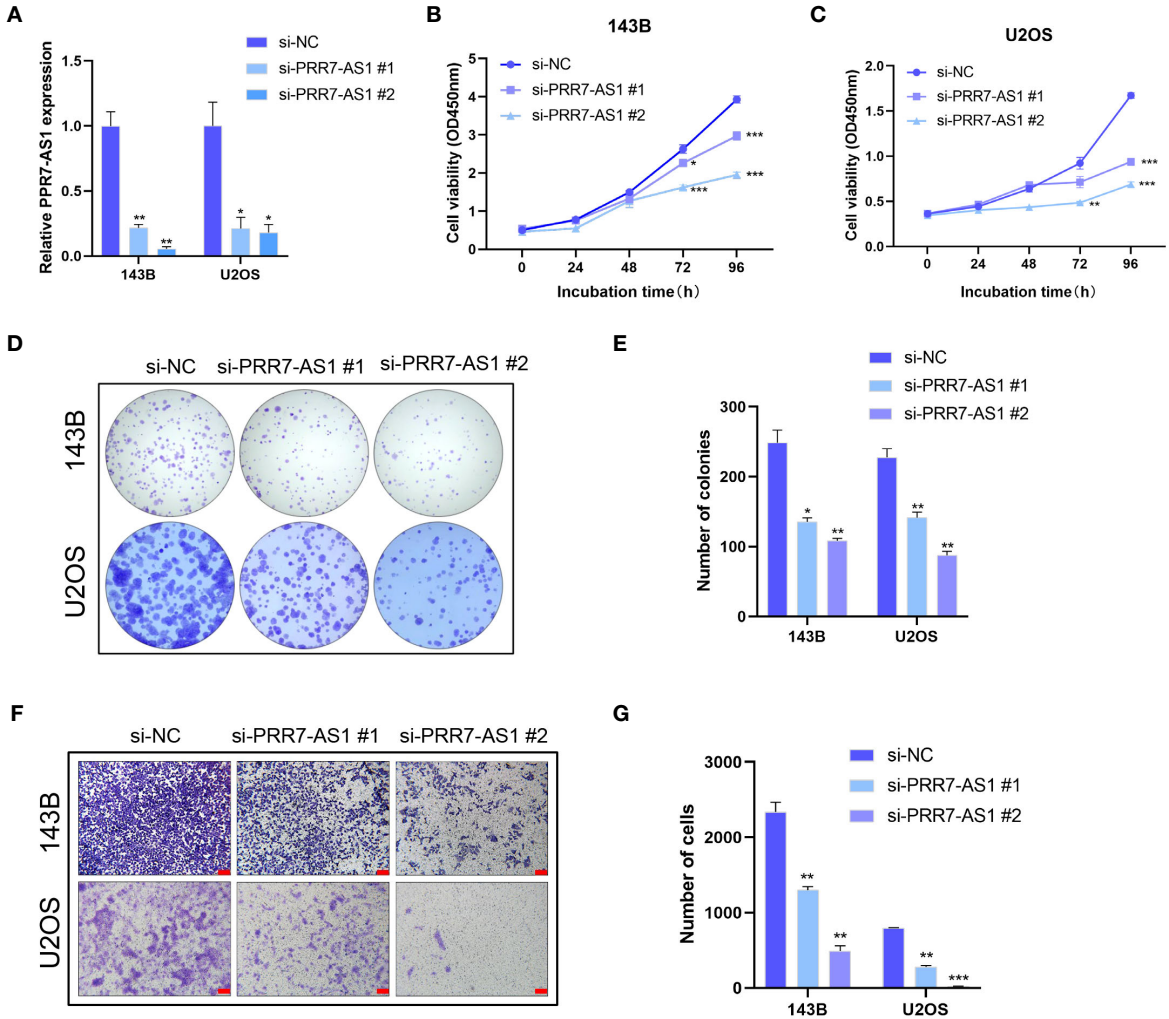
Effects of PRR7-AS1 on OS cells proliferation and migration capacity in vitro. **(A)** Relative PRR7-AS1 expression in 143B and U2OS cells transfected with negative control siRNA (si-NC) or siRNAs targeting PRR7-AS1 (si-PRR7-AS1 #1 and #2) for 48 h (n = 3 for each group). **(B, C)** Cell viability was assessed using a CCK8 assay in 143B and U2OS cells transfected with si-NC or si-PRR7-AS1 (n = 6 for each group). *P < 0.05, **P < 0.01 and ***P < 0.001. **(D, E)** Colony formation assays were performed to determine the proliferative ability of si-PRR7-AS1-transfected 143B and U2OS cells (n = 3 for each group). *P < 0.05, **P < 0.01. **(F, G)** Transwell assays were performed to investigate the migration capacity of 143B and U2OS cells after PRR7-AS1 knockdown for 48 h (n = 3 for each group). Scale bar= 100 mm. **P < 0.01, ***P < 0.001.

The transfection efficiency of si-PRR7-AS1 were verified by RT-qPCR (**Figure 2A**). Both CCK8 and colony formation assays found that knockdown of PRR7-AS1 significantly inhibited the proliferative capacity of 143B and U2OS cells (**Figures 2B–E**). In addition, transwell assays revealed a lower migration capacity of OS cells transfected with si-PRR7-AS1 than si-NC (**Figures 2F, G**). These findings indicated that PRR7-AS1 promotes the proliferation and migration of OS cells *in vitro*.

### 3.3 PRR7−AS1 promotes tumor growth and metastasis of OS *in vivo*

**Figure 3:**
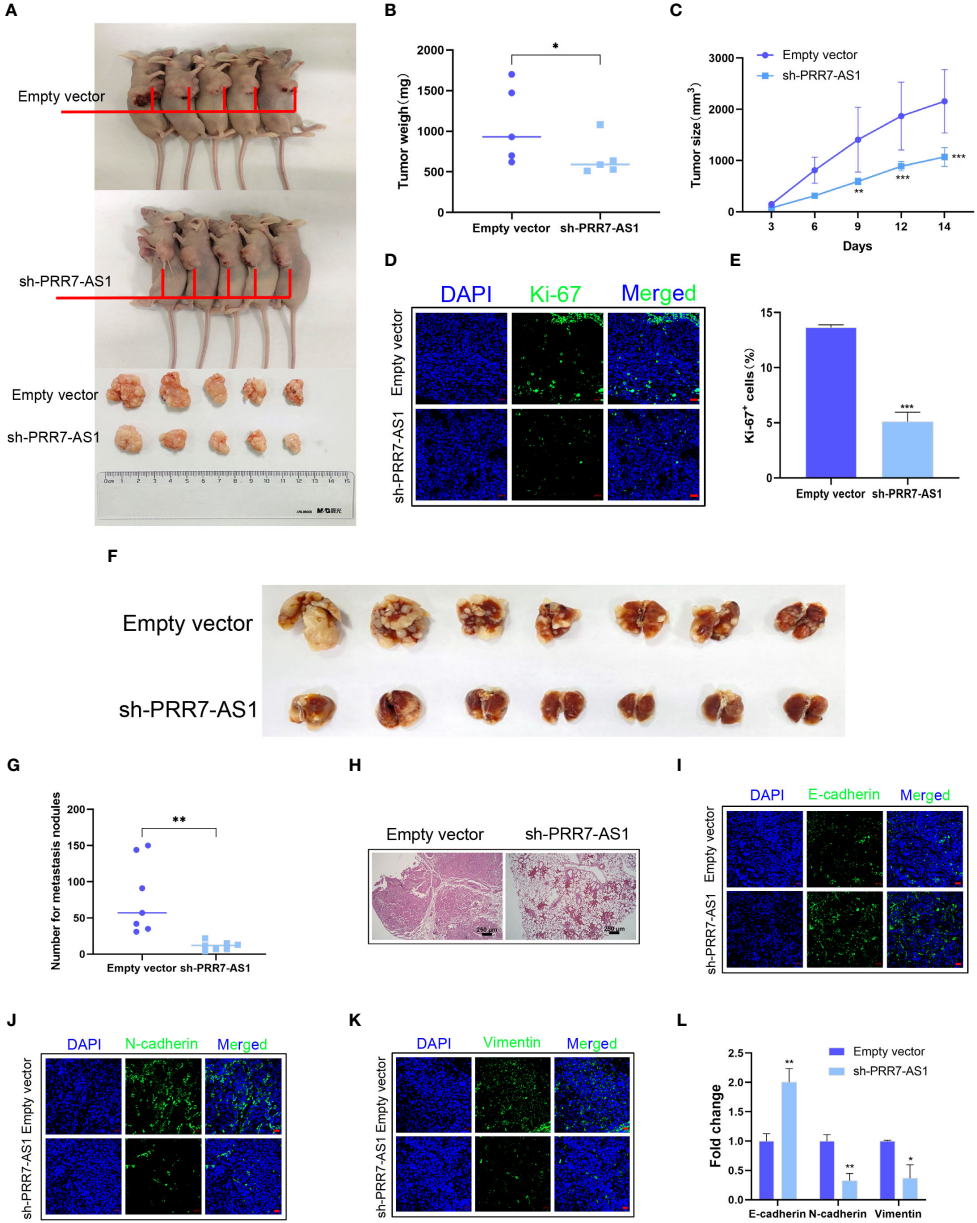
Effects of PRR7-AS1 on OS tumorigenesis and metastasis in vivo. 143B cells stably expressing shPRR7-AS1 or empty vector (control) were injected into nude mice (n=5). **(A)** Tumors were isolated from the nude mice and photographed, and tumor weights were measured **(B)**. *P < 0.05 **(C)** Tumor volumes were calculated every 3 days after injection. **P < 0.01, ***P < 0.001. **(D, E)** Immunostaining for the Ki-67+ cells in tumor sections (n = 3 for each group). Scale bar = 50 mm. ***P < 0.001. **(F)** 143B cells were transfected with sh-PRR7-AS1 or empty vector for 48 h and injected into the tail vein of nude mice (n = 7). After 2 months, lung tissues were removed and photographed. **(G)** The number of lung nodules was counted. **(H)** H&E staining of mouse lung tissues. **P < 0.01. **(I–K)** Immunofluorescence staining of E-cadherin, Ncadherin and vimentin, bar = 50 mm. **(L)** Quantification of **(D–F)**. *P < 0.05, **P < 0.01.

A xenograft model in nude mice was established to explore the *in vivo* function of PRR7-AS1. During the experimental period, tumor growth was significantly lower in the sh-PRR7-AS1 group compared to the controls. After 2 weeks, mice were sacrificed, and OS tissues were collected. Compared to the control group, both the volume and weight of OS tissues were significantly lower in the sh-PRR7-AS1 group than empty vector group (**Figures 3A–C**). In addition, the lower Ki-67-positive cells was shown in the sh-PRR7-AS1 group too (**Figures 3D, E**).

We established an OS lung metastasis model in nude mice through injecting 143B cells into the mouse tail vein as previously reported (28). Compared to the controls, the number of lung metastases was significantly lower in nude mice injected with 143B cells transfected with sh-PRR7-AS1 (**Figures 3F, G**). The same results were obtained with H&E staining (**Figure 3H**). Furthermore, increased E-cadherin as well as decreased N-cadherin and vimentin expression were obviously in the sh-PRR7-AS1 group (**Figure 3I–L**).

Collectively, these findings demonstrated that PRR7-AS1 promotes the proliferation and metastasis of OS cells *in vivo*.

### 3.4 PRR7−AS1 interacts with RNF2 in OS cells

**Figure 4:**
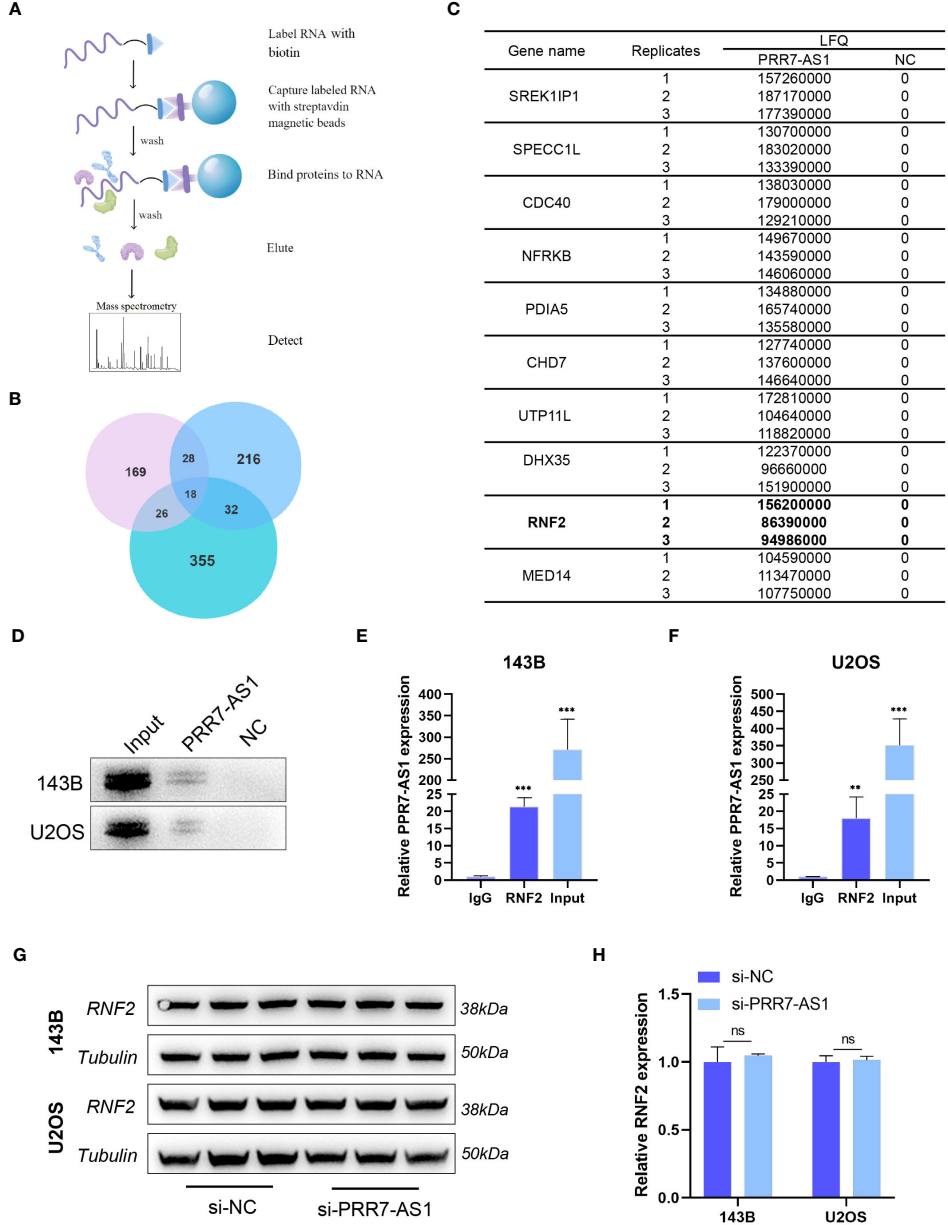
PRR7-AS1 interacts with RNF2. **(A)** Flow chart of the RNA pull-down assays. **(B)** Venn diagrams of proteins identified by mass spectrometry from three independent RNA pull-down assays. **(C)** The list of top 10 proteins. **(D)** Biotinylated PRR7-AS1 RNAs were incubated with 143B or U2OS cell lysates. The RNA–protein complexes were subjected to western blot analysis with an anti-RNF2 antibody. The antisense strand of PRR7-AS1 was used as the negative control. **(E, F)** RNA immunoprecipitation (RIP) assays for PRR7-AS1 binding to RNF2 in 143B and U2OS cells lysates. Rabbit IgG was included as the negative control for immunoprecipitation (n = 3). **P < 0.01 and ***P < 0.001. **(G)** The protein level of RNF2 were detected in 143B and U2OS transfected with si-NC and si-PRR7-AS1 using western blotting (n = 3). **(H)** Quantification of **(G)**. ns, not statistically significant.

To further explore the molecular mechanism underlying the carcinogenic activity of PRR7-AS1 in OS, an RNA pull-down LC-MS/MS assay was performed to identify potential proteins that interact with lncRNA PRR7-AS1 in OS cells (**Figure 4A**).

According to the label-free quantitation (LFQ) method, proteomic analysis identified 18 overlapping proteins from three independent RNA pull-down experiments (**Figure 4B**; **Table S3**). **Figure 4C** shows the top 10 proteins listed from high to low. Among them, RNF2 (ring finger protein 2) caught our attentions. As a member of the polycomb group (PcG), RNF2 is widely involved in tumor development through epigenetic regulation (29). RNA pull-down and RIP experiments in 143B and U2OS cells further confirmed the interaction between PRR7-AS1 and RNF2 (**Figures 4D–F**). Strikingly, it was noticed that RNF2 expression levels remained unaltered after knockdown of PRR7-AS1 compared to control (**Figures 4G, H**). These findings suggest that PRR7-AS1 only binds RNF2, but does not affect its expression.

### 3.5 RNF2 is involved in gene regulation and promotes the proliferation and migration of OS *in vitro*

**Figure 5:**
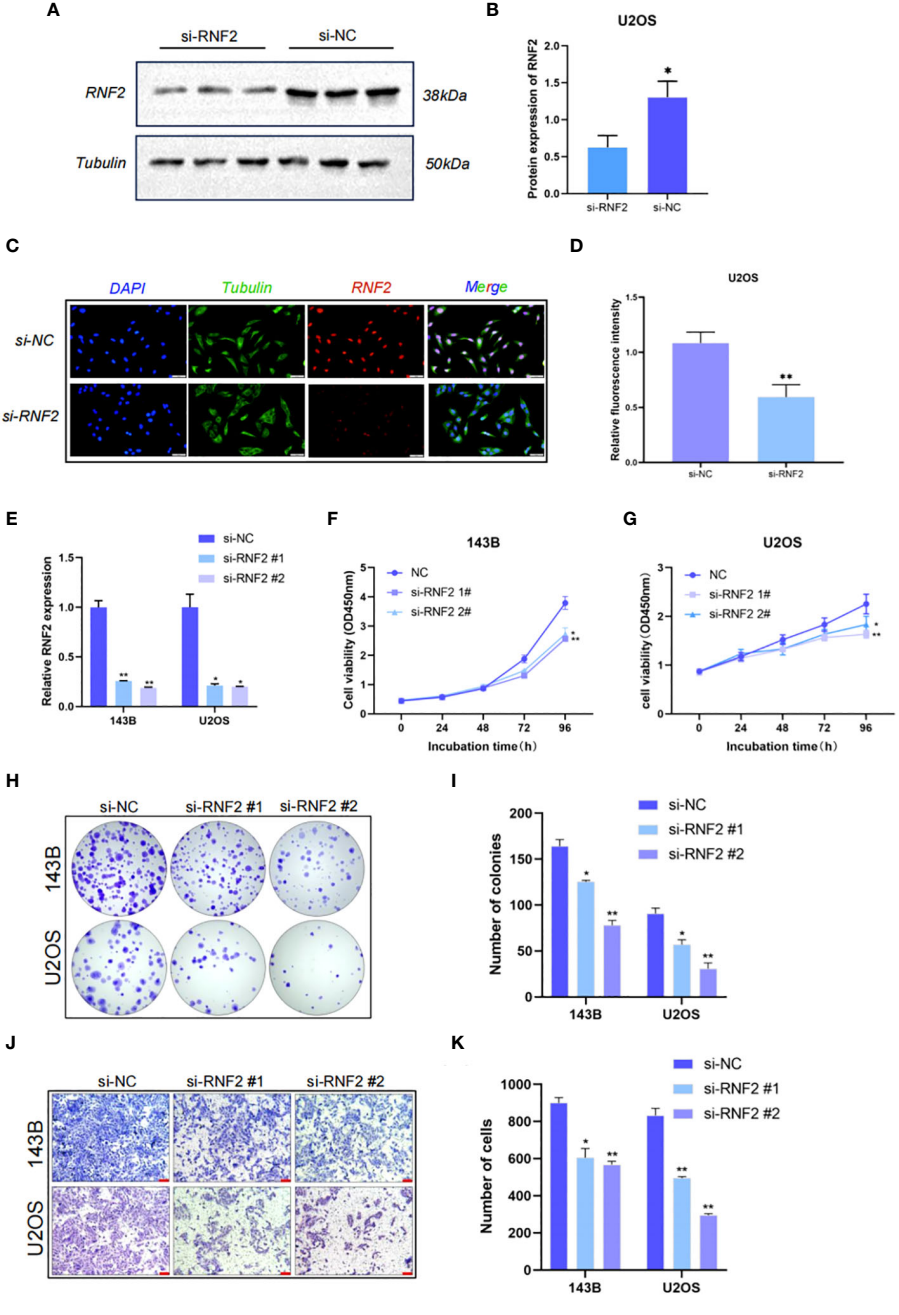
RNF2 is involved in gene regulation and promotes the proliferation and migration of OS in vitro. **(A)** Western blot analysis of RNF2 protein after the treatment with si-RNF2 or si-NC. Beta-tubulin protein was used as an internal control. **(B)** Quantification of RNF2 protein levels in **(A)**, n = 3. *P < 0.05. **(C)** Immunostaining of RNF2 in U2OS treated with si-NC or si-RNF2. RNF2 marker proteins are labeled in red, Tubulin marker proteins are labeled in green and nucleus are labeled in blue. **(D)** Quantitative immunofluorescence of RNF2 marker proteins in **(C)**, n = 3. **P < 0.01. **(E)** RT-qPCR analysis was used to detect the relative RNF2 mRNA expression levels after transfection with si-RNF2 (n = 3). *P < 0.05, **P < 0.01. **(F–K)** CCK8 (n = 6) **(F, G)**, colony formation (n = 3) **(H, I)** and transwell (n = 3, scale bar = 100 mm) **(J, K)** assays were used to assess the proliferative and migration capacity of 143B and U2OS cells transfected with si-NC or si-RNF2. *P < 0.05, **P < 0.01.

We selected U2OS cells for western blotting and immunofluorescence experiments of RNF2. The protein expression of RNF2 was significantly reduced after knockdown of RNF2 (**Figures 5A, B**). Immunofluorescence analysis showed that RNF2 protein was primarily localized in the nucleus rather than the cytoplasm and could be significantly knocked down (**Figures 5C, D**), which further verified that RNF2 protein was involved in gene regulation. To explore the oncogenic features of RNF2 in OS, we synthesized two RNF2 siRNAs and verified their transfection efficiency (**Figure 5E**). Knockdown of RNF2 significantly reduced cell viability and the number of colonies in 143B and U2OS cells, indicating inhibition of proliferation (**Figures 5F–I**). Moreover, the number of migratory cells was reduced in OS cells transfected with si-RNF2 compared to si-NC (**Figures 5J, K**). Therefore, these findings suggested that RNF2 facilitates the proliferation and migration of OS cells *in vitro*.

### 3.6 “PRR7-AS1-RNF2” inhibits the transcription of MTUS1 through histone H2A lysine 119 monoubiquitin

**Figure 6:**
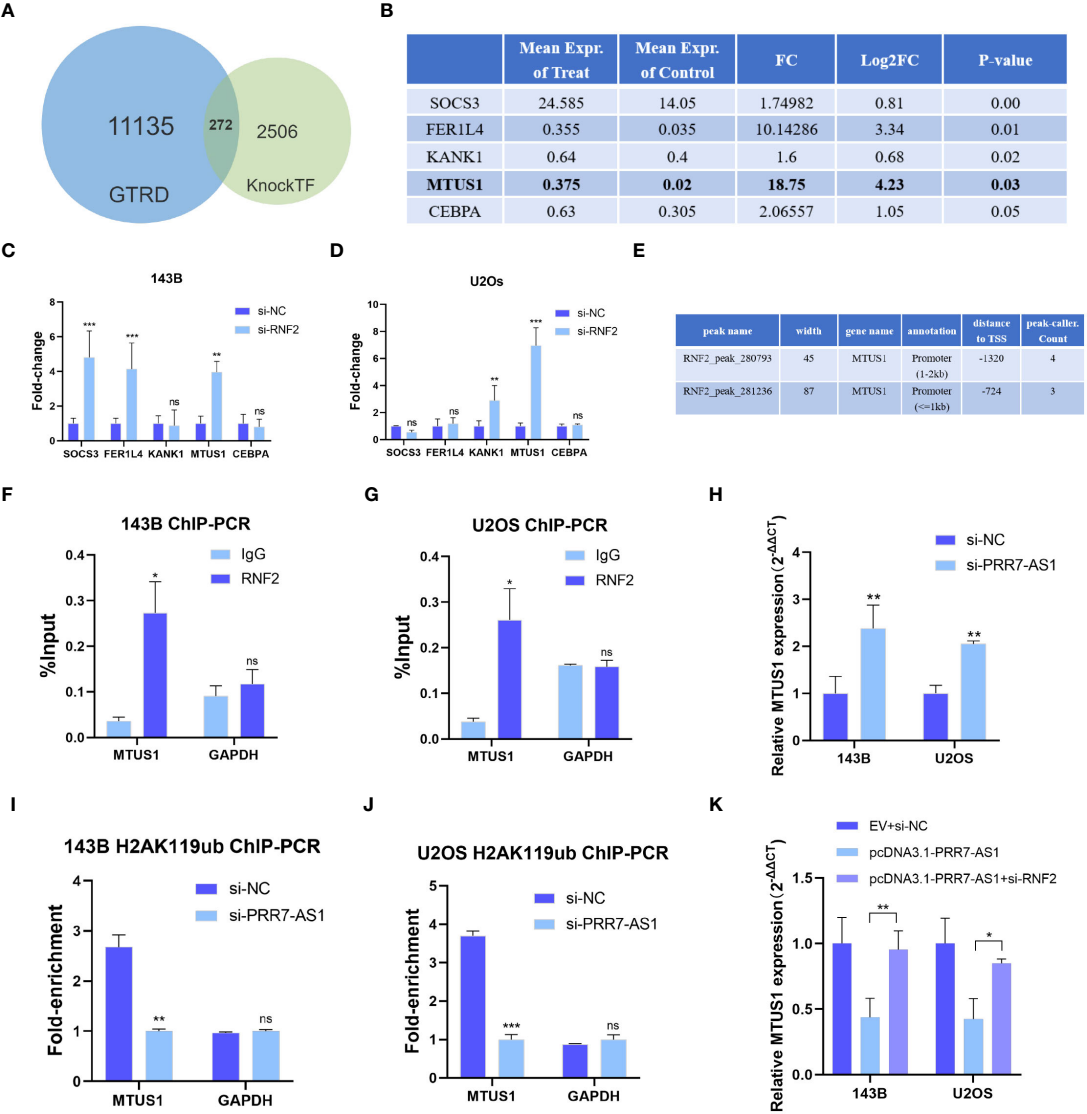
RNF2 inhibits the transcription of MTUS1. **(A)** Venn diagram analysis of putative RNF2 targets from the GTRD and KnockTF database. **(B)** Gene list of selected RNF2 targets (data from KnockTF). **(C, D)** RT-qPCR analysis was used to assess the mRNA levels of candidate genes after RNF2 knockdown in OS cells. **P < 0.01, ***P < 0.001. ns, not statistically significant. **(E)** Putative binding peaks of RNF2 on MTUS1 (data from GTRD). **(F, G)** ChIP-qPCR of RNF2-associated DNA sequences from the RNF2- binding region of the MTUS1 promoter in OS cells. The GAPDH gene was used as a negative control. **(H)** RT-qPCR analysis was used to assess the mRNA levels of MTUS1 after PRR7-AS1 knockdown in OS cells. **(I, J)** ChIP-PCR of H2AK119ub-associated DNA sequences in the putative RNF2- binding region of the MTUS1 promoter in OS cells treated with si-NC and si-PRR7-AS1. The GAPDH gene was used as a negative control. **(K)** Relative MTUS1 mRNA level was tested in 143B and U2OS transfected with EV + si-NC, pcDNA3.1-PRR7-AS1, and pcDNA3.1-PRR7-AS1 + si-RNF2 via RTqPCR (n = 3). *P < 0.05, **P < 0.01 and ***P < 0.001. ns, not statistically significant.

It has been reported that RNF2 contributes to inhibit gene transcription as a component of the polycomb repressive complex 1 (PRC1) (30). The KnockTF database predicted a total of 14,096 differentially expressed genes after knockdown of RNF2, and 2,778 genes were upregulated (|FC| >1.5). Through screening the ChIP-Seq datasets extracted from the publicly available Sequence Read Archive (SRA), GEO and ENCODE databases by GTRD, a total of 11,407 genes were identified to bind the RNF2 transcription factor, including 23,655 binding peaks. Venn diagram analysis yielded 272 candidate genes that not only bound to RNF2 but also were regulated by RNF2 (**Figure 6A**). Five of the target genes have been previously reported to have an oncogenic effect on OS cells (**Figure 6B**). RT-qPCR were performed to further confirm the regulatory effect of RNF2 on these target genes. MTUS1 (microtubule associated scaffold protein 1) was significantly upregulated in both 143B and U2OS cells after knockdown of RNF2 (**Figures 6C, D**). Therefore, RNF2 might target MTUS1 in OS cells.

RNF2 exerts its biological function in silencing target genes through histone H2A lysine 119 monoubiquitin (H2AK119ub) (31). The RNF2-binding region predicted by the GTRD falls from -2000 to +100 bp of the MTUS1 transcription start point, which is its promoter region (**Figure 6E**). ChIP-qPCR results showed that RNF2 was significantly recruited in this region (**Figures 6F, G**). Moreover, MTUS1 mRNA expression was upregulated after PRR7-AS1 silencing in 143B and U2OS (**Figure 6H**). Knockdown of PRR7-AS1 reduced the recruitment of H2AK119ub to the MTUS1 promoter (**Figures 6I, J**). Taken together, these findings demonstrated that PRR7-AS1 is required for RNF2 to inhibit the transcription of MTUS1 through H2AK119ub. Furthermore, it is obvious that overexpressing PRR7-AS1 decreased the MTUS1 mRNA level and knockdown of RNF2 reverses this decrease (**Figure 6K**). This finding demonstrated that PRR7-AS1 inhibit the expression of MTUS1 dependent on RNF2.

### 3.7 “PRR7-AS1-RNF2” promotes the cell proliferation and migration depend on MTUS1 in OS cells

**Figure 7:**
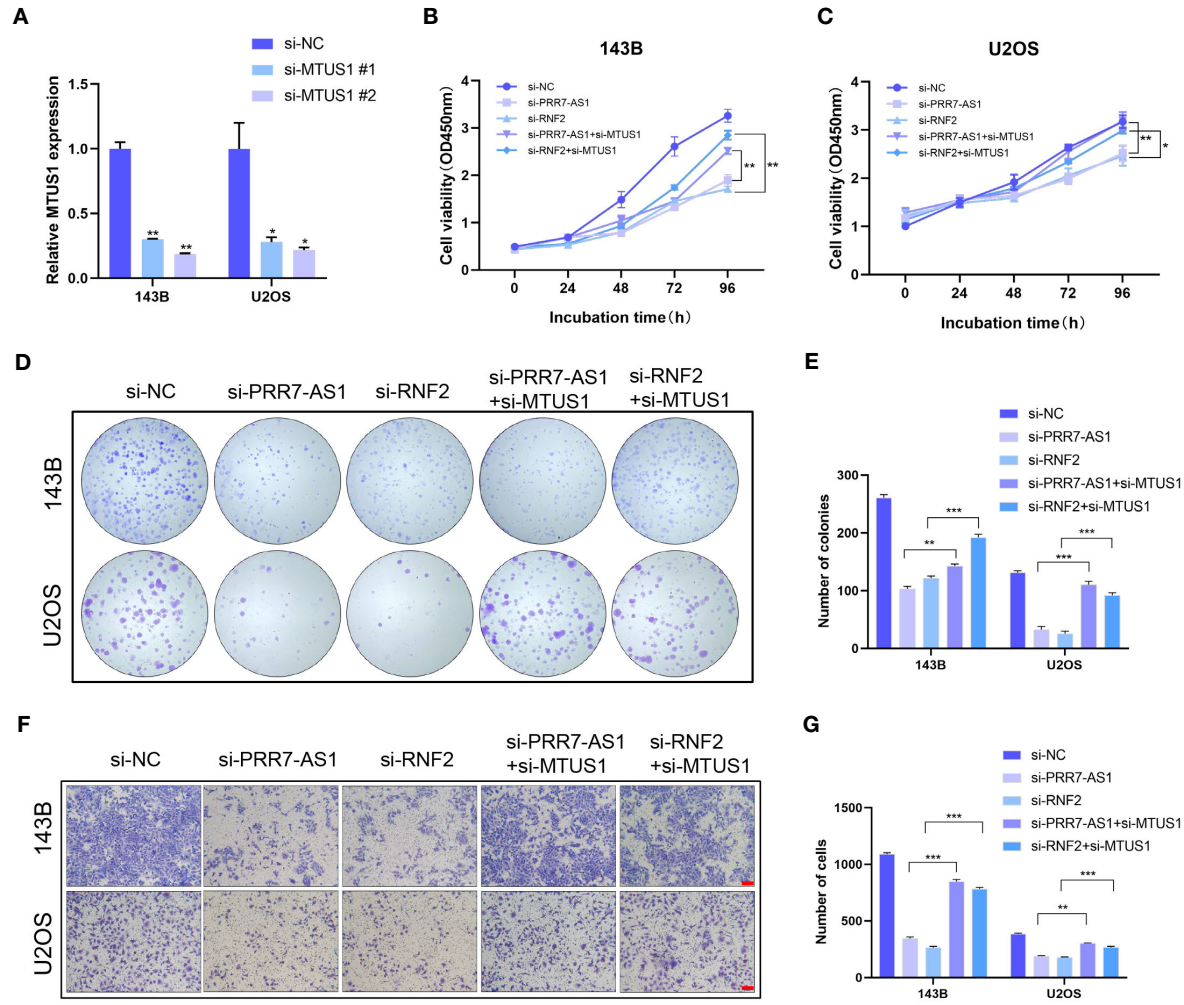
Knockdown of MTUS1 reverses the regulatory effect of PRR7-AS1 and RNF2 on OS cell behaviors. **(A)** Relative expression levels of MTUS1 in 143B and U2OS cells transfected with si-NC, si-MTUS1 were detected by RT-qPCR. *P < 0.05, **P < 0.01. **(B–E)** After co-transfection with si-MTUS1 and si-PRR7-AS1 or si-RNF2 for 48 h, the proliferative ability of OS cells was detected using CCK8 assays (n = 6) and colony formation assay (n = 3). *P < 0.05, **P < 0.01 and ***P < 0.001. **(F)** The cell migration in 143B and U2OS cells was detected by Transwell assays, n = 3, scale bar = 100 mm. **(G)** Quantification of **(F)**. **P < 0.01, ***P < 0.001.

MTUS1 is a tumor suppressor gene that has been validated to inhibit the proliferation and migration of OS cells. The transfection efficiency of si-MTUS1 were verified by RT-qPRCR (**Figure 7A**), si-MTUS1 2# was used in the following experiments. 143B and U2OS cells were co-transfected with si-MTUS1 and si-PRR7-AS1/si-RNF2. Interestingly, the proliferative and migration capacities of co-transfected cells were significantly higher than those only transfected with si-PRR7-AS1 or si-RNF2 (**Figures 7B–G**). These findings indicated that knockdown of MTUS1 reverses the inhibited proliferation and migration of OS cells caused by knockdown of PRR7-AS1 and RNF2.

## 4 Discussion

OS is a common malignant tumor that mainly occurs in children and adolescents (32), and it is characterized by rapid progression, high metastasis rate and high mortality. At present, the precise pathogenesis of OS has not been fully elucidated. Surgery combined with adjuvant chemoradiotherapy is the main treatment of OS. Because early diagnosis is of significance to improve the prognosis, it is urgent to identify effective biomarkers for the diagnosis and management of OS. LncRNAs have been suggested to have clinical potential, and they are involved in cancer development (11, 12). In the present study, we first identified that PRR7-AS1 was upregulated in OS tissues, and high expression of PRR7-AS1 indicated a poorer prognosis in OS through bioinformatics analysis. Subsequently, loss-function-assays demonstrated lncRNA PRR7-AS1 is a vital oncogene involved in the development of OS.

The biological functions of lncRNAs are diverse and complex with the involvement of multiple mechanisms, and lncRNAs regulate signaling pathways and serve as molecular decoys, guiding molecules and structural scaffolds (33, 34). In the present study, RNA pull-down was performed to search for proteins that interact with RPP7-AS1. Among them, increasing evidence has shown that RNF2 contributes to influence clinical characteristics of many types of cancers, including hepatocellular carcinoma, melanoma, prostate cancer, breast cancer, pancreatic cancer, gastric cancer and bladder urothelial carcinoma (29, 35, 36). RNA-pulldown-western-blot and RIP-PCR were verified this interaction. Through Western blot analysis, we suggest that PRR7-AS1 does not affect the expression of RNF2, which is likely to recruit RNF2 to their target sites. In addition, immunofluorescence analysis confirmed that RNF2 proteins were mainly localized in the nucleus, suggesting that RNF2 appears to be involved in gene regulation in OS cells.

The loss-function-assay has confirmed that RNF2 promoted the proliferation and migration of OS cells. We hypothesized that RNF2 forms a protein complex with PRR7-AS1, which further localizes to a specific DNA sequence to regulate the transcription of downstream genes. We next searched for downstream targets of RNF2 using the GTRD and KnockTF database. Through collecting uniformly processed ChIP-seq data from the SRA, GEO and ENCODE databases, GTRD identifies transcription factor binding sites (TFBS) and their motifs via peak calling. KnockTF is a comprehensive human gene expression profile database with TF knockdown/knockout (KnockTF), which provides a human gene expression profile dataset associated with TF knockdown/knockout and annotates TFs and their target genes in tissues or cells. Because the RNF2 oncogene exerts the inhibitory effect on gene transcription through H2AK119ub (37, 38), a total of 272 candidate genes who upregulated after RNF2 knockdown were identified.

Based on these findings and a literature review, MTUS1 was selected as the potential downstream target of RNF2, which has been demonstrated to be a tumor suppressor gene. MTUS1 is located on the human chromosome antisense strand 8p22, and it encodes a protein containing a C-terminal domain that interacts with the angiotensin II (AT2) receptor (39). Abnormally expressed MTUS1 is closely linked with colorectal cancer and prostate cancer (40, 41). In addition, MTUS1 promotes the development of OS through regulating the ERK/EMT signaling pathway (42). Knockdown of either PRR7-AS1 or RNF2 in OS cells significantly upregulated MTUS1, which indicated an inhibitory effect of the protein complex formed by PRR7-AS1 and RNF2 on the downstream target. In addition, knockdown of PRR7-AS1 significantly reduced the enrichment of H2AK119ub, suggesting that through binding RNF2, PRR7-AS1 induces the binding of RNF2 in the promoter region of the MTUS1 downstream gene, thus triggering the monoubiquitin of H2AK119 and inhibiting the transcription of MTUS1. Vitally, the capacity of RNF2 to bind and suppress the transcription of MTUS1 depended on PRR7-AS1, and PRR7-AS1 suppress the transcription of MTUS1 through targeting RNF2. Furthermore, this study revealed that knockdown of MTUS1 reversed the regulatory effect of PRR7-AS1 and RNF2 on OS cells behaviors, validating the anti-cancer role of MTUS1 in OS.

**Figure 8:**
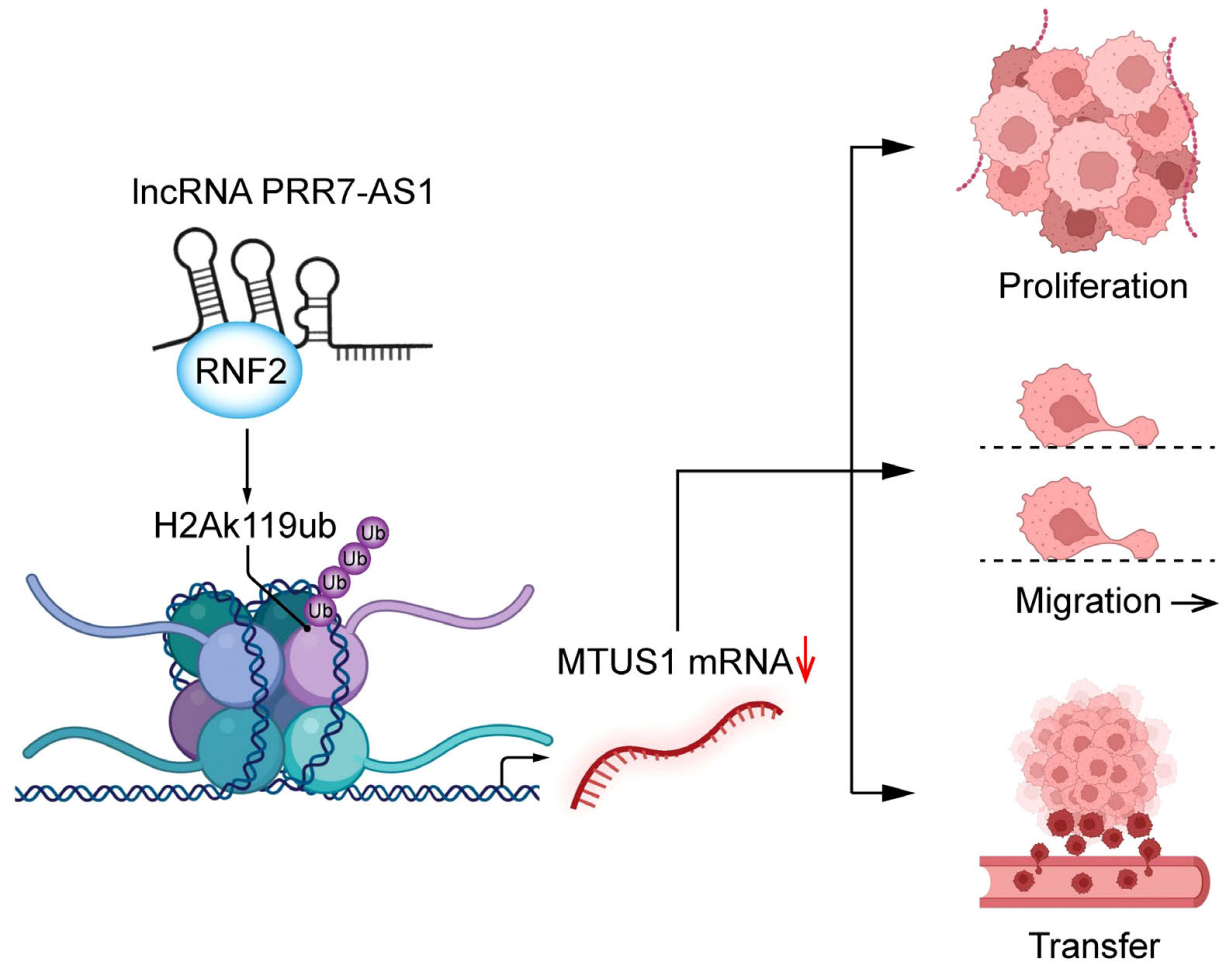
Schematic diagram of lncRNA PRR7-AS1 functions to promote tumor proliferation, migration and metastasis in OS cells.

Taken together, our findings demonstrated that PRR7-AS1 is upregulated in OS tissues and is closely linked with the survival and prognosis of OS patients. PRR7-AS1 promotes the proliferation and migration of OS cells by binding RNF2, thereby inhibiting the transcription of MTUS1 through H2AK119ub (**Figure 8**). In summary, the PRR7-AS1/RNF2/MTUS1 axis promotes proliferation and migration of OS cells. Therefore, our study provides a novel biomarker that may be utilized in a lncRNA-guided therapeutic strategy for OS.

## Data availability statement

The datasets presented in this study can be found in online repositories. The names of the repository/repositories and accession number(s) can be found in the article/**Supplementary Material**.

## Ethics statement

The studies involving humans were approved by Ethics Committee of Suzhou Municipal Hospital. The studies were conducted in accordance with the local legislation and institutional requirements. The participants provided their written informed consent to participate in this study. The animal study was approved by Ethics Committee of Animal Experiments of Nanjing Medical University. The study was conducted in accordance with the local legislation and institutional requirements.

## Author contributions

GC-X: Investigation, Data curation, Writing – original draft, Formal analysis. XJ-F: Investigation, Validation, Visualization. HA-Q: Investigation, Data curation, Methodology. YX: Investigation, Data curation, Writing – review & editing. WY-H: Investigation, Data curation. LS-Y: Investigation. SC: Supervision, Project administration. ZT-M: Supervision, Project administration, Funding acquisition. SJ: Conceptualization, Funding acquisition. All authors contributed to the article and approved the submitted version.

## Supplementary material

Supplementary Figure 1 Kaplan–Meier curves of differentially expressed lncRNAs.
