# Supplementary figures and images for: Long non-coding RNA PRR7-AS1 promotes osteosarcoma progression via binding RNF2 to transcriptionally suppress MTUS1
Source: Front Oncol. 2023 Nov 16;13:1227789. doi: 10.3389/fonc.2023.1227789 (PMC10687407; doi:10.3389/fonc.2023.1227789)

overall survival

AC010776.2

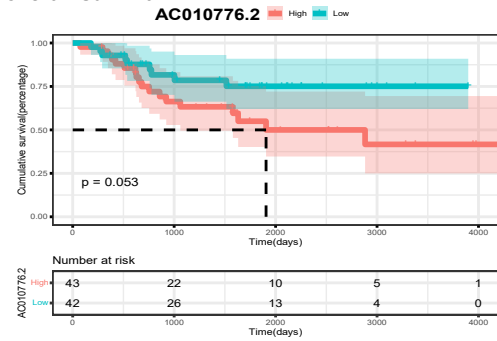

AC037198.1

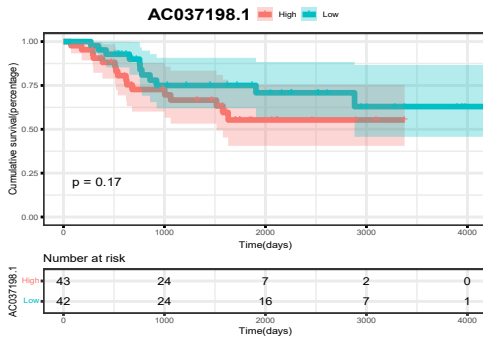

AC092691.1

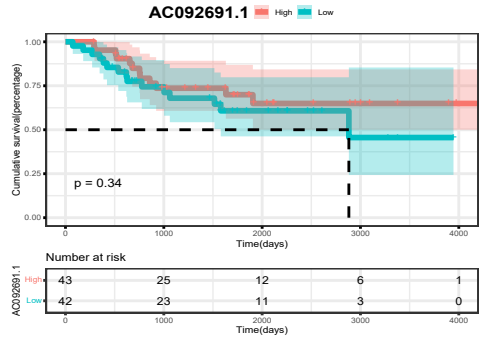

AC110597.1

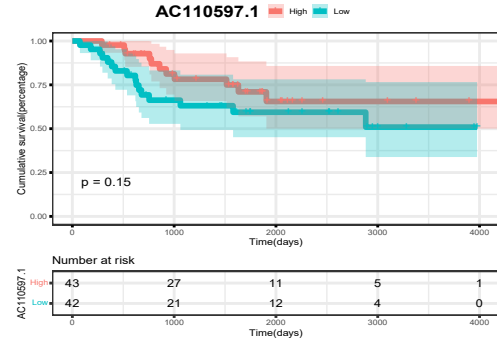

AC139769.2

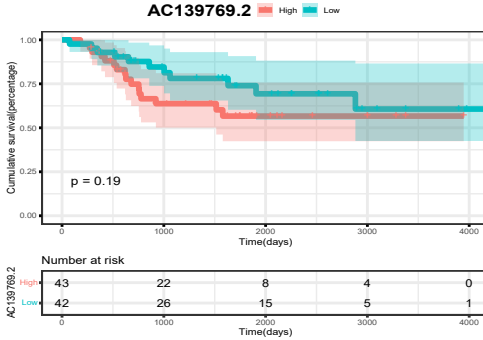

AC156455.1

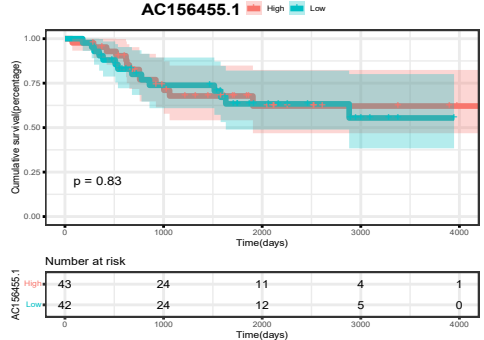

AL365181.3

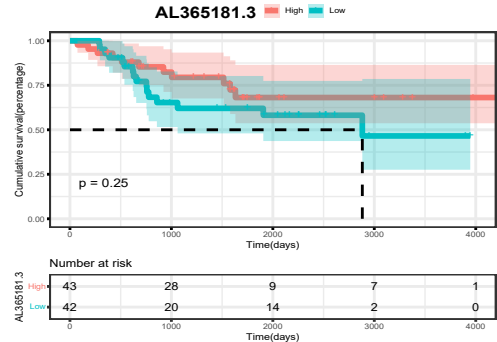

LINC01111

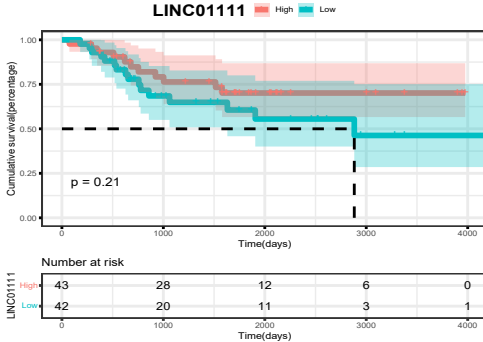

LINC01116

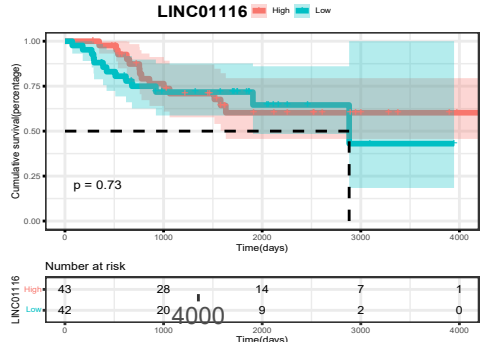

LINC01139

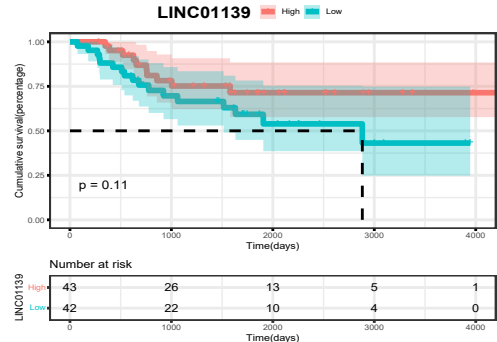

LINC02802

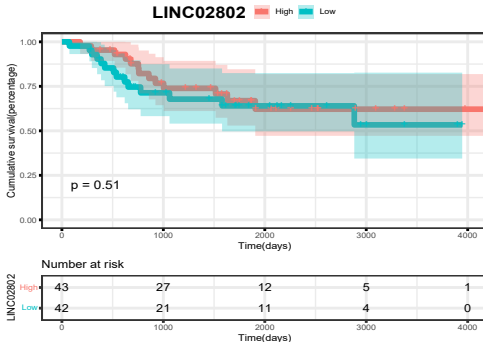

LNCOG

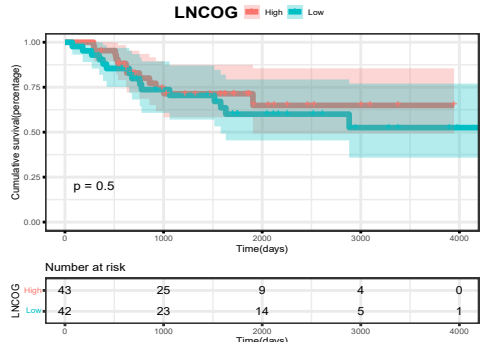

MIR155HG

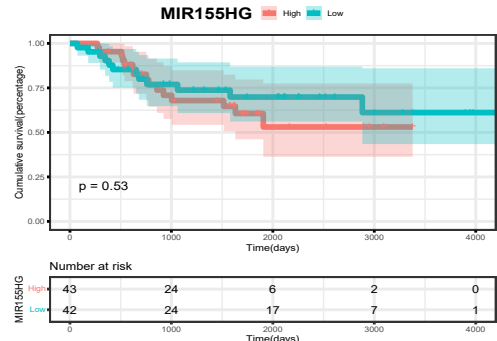

PVT1

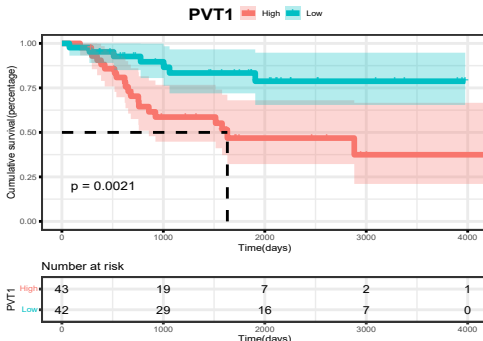

RGMB-AS1

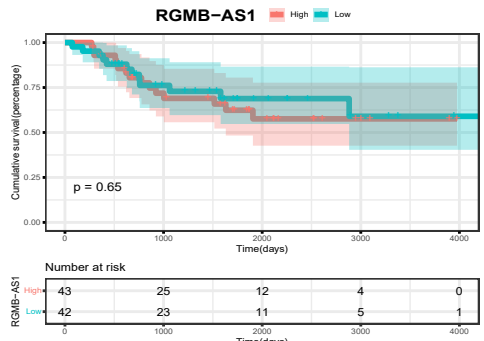

SNHG4

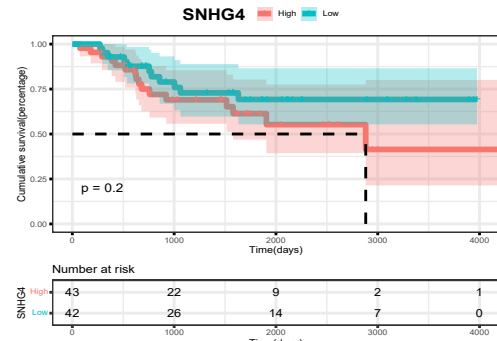

TMCC1-AS1

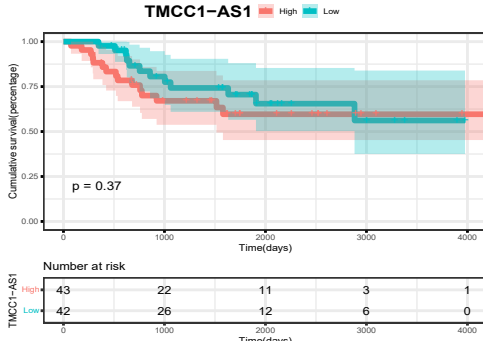

ZFAS1

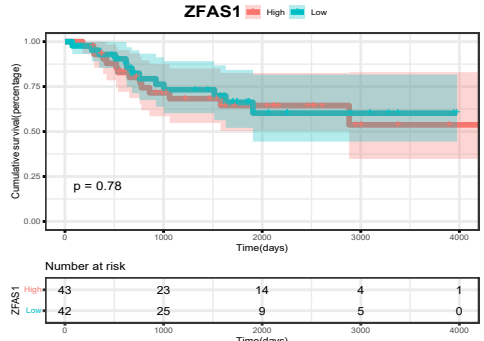

Supplement: Supplementary Figure 1 — Kaplan–Meier curves of differentially expressed lncRNAs. [file DataSheet_1.pdf]
