# Supplementary material for: Long non-coding RNA PRR7-AS1 promotes osteosarcoma progression via binding RNF2 to transcriptionally suppress MTUS1
Source: Front Oncol. 2023 Nov 16;13:1227789. doi: 10.3389/fonc.2023.1227789 (PMC10687407; doi:10.3389/fonc.2023.1227789)
Supplement: Supplementary file 2 [file Table_1.docx]

Table S1. The sequence of siRNAs in this study.

| siRNAs | Sequences |
| --- | --- |
| si-NC | 5’-UUCUCCGAACGUGUCACGU -3’ |
| si-PRR7-AS1 1# | 5’-CCAACUGACGAACAGGUAU-3’; |
| si-PRR7-AS1 2# | 5’-CCAUCUUCGUAGCUUGCUU -3’; |
| si-PRR7-AS1 3# | 5’-GCUCCAGUGGGCCUCAUUA -3’; |
| si-RNF2 1# | 5’-GGAUCAACAAGCACAAUAA3’; |
| si-RNF2 2#  si-MTUS1 1#  si-MTUS1 2# | 5’-CCAGUUCACUGUAUUAAAU-3’;  5’-GCCCAAGACAUGACUUACA-3’;  5’-GGAGUUAGAAAGCCUGAAA-3’; |
