# Supplementary material for: Long non-coding RNA PRR7-AS1 promotes osteosarcoma progression via binding RNF2 to transcriptionally suppress MTUS1
Source: Front Oncol. 2023 Nov 16;13:1227789. doi: 10.3389/fonc.2023.1227789 (PMC10687407; doi:10.3389/fonc.2023.1227789)
Supplement: Supplementary file 3 [file Table_2.docx]

Table S2. The list of primers sequences in this study.

| Primers | Sequences |
| --- | --- |
| PRR7-AS1-F | 5’-GCTAGGAACACGGAGAGAG-3’; |
| PRR7-AS1-R | 5’-CCTGAGTGACTGCCTCACAT -3’; |
| MTUS1-F | 5’-CTCGTCGCTGTCACCTTGAA -3’;; |
| MTUS1-R | 5’-CAACCCTATTCCACCCGCTT -3’; |
| RNF2-F | 5’- CAGGAGCCGCAATGTCTCA-3’; |
| RNF2-R  18sRNA -F  18sRNA -R | 5’-GCCATCTGTTATTGCCTCCTGA  5’-AAACGGCTACCACATCCAAG-3’;  5’-CCTCCAATGGATCCTCGTTA-3’. |
