# Supplementary material for: Long non-coding RNA PRR7-AS1 promotes osteosarcoma progression via binding RNF2 to transcriptionally suppress MTUS1
Source: Front Oncol. 2023 Nov 16;13:1227789. doi: 10.3389/fonc.2023.1227789 (PMC10687407; doi:10.3389/fonc.2023.1227789)
Supplement: Supplementary file 4 [file Table_3.docx]

Table S3. Putative inter-actors identified from three independent RNA pull-down assays in this study

| Protein names | Gene names | LFQ intensity | | | | | |
| --- | --- | --- | --- | --- | --- | --- | --- |
|  |  | Ctr | | | PRR7-AS1 | | |
| Protein SREK1IP1 | SREK1IP1 | 0 | 0 | 0 | 157260000 | 187170000 | 177390000 |
| Cytospin-A | SPECC1L; SPECC1L-ADORA2A | 0 | 0 | 0 | 130700000 | 183020000 | 133390000 |
| Pre-mRNA-processing factor 17 | CDC40 | 0 | 0 | 0 | 138030000 | 179000000 | 129210000 |
| Nuclear factor related to kappa-B-binding protein | NFRKB | 0 | 0 | 0 | 149670000 | 143590000 | 146060000 |
| Protein disulfide-isomerase A5 | PDIA5 | 0 | 0 | 0 | 134880000 | 165740000 | 135580000 |
| Chromodomain-helicase-DNA-binding protein 7 | CHD7 | 0 | 0 | 0 | 127740000 | 137600000 | 146640000 |
| Probable U3 small nucleolar RNA-associated protein 11 | UTP11L | 0 | 0 | 0 | 172810000 | 104640000 | 118820000 |
| Probable ATP-dependent RNA helicase DHX35 | DHX35 | 0 | 0 | 0 | 122370000 | 96660000 | 151900000 |
| E3 ubiquitin-protein ligase RING2 | RNF2 | 0 | 0 | 0 | 156200000 | 86390000 | 94986000 |
| Mediator of RNA polymerase II transcription subunit 14 | MED14 | 0 | 0 | 0 | 104590000 | 113470000 | 107750000 |
| INO80 complex subunit C | INO80C | 0 | 0 | 0 | 99928000 | 121710000 | 103960000 |
| HMG domain-containing protein 4 | HMGXB4 | 0 | 0 | 0 | 92663000 | 119020000 | 77093000 |
| Nucleosome-remodeling factor subunit BPTF | BPTF | 0 | 0 | 0 | 84595000 | 113380000 | 74855000 |
| AT-rich interactive domain-containing protein 2 | ARID2 | 0 | 0 | 0 | 67932000 | 74167000 | 88748000 |
| Negative elongation factor E | NELFE | 0 | 0 | 0 | 55401000 | 62215000 | 55891000 |
| U3 small nucleolar ribonucleoprotein protein IMP4 | IMP4 | 0 | 0 | 0 | 61962000 | 38077000 | 68202000 |
| PHD and RING finger domain-containing protein 1 | PHRF1 | 0 | 0 | 0 | 51182000 | 54527000 | 47467000 |
| Elongator complex protein 3 | ELP3 | 0 | 0 | 0 | 50875000 | 49476000 | 49322000 |
